# Pedometer-determined physical activity and active transport in girls

**DOI:** 10.1186/1479-5868-5-2

**Published:** 2008-01-11

**Authors:** Elizabeth K Duncan, J Scott Duncan, Grant Schofield

**Affiliations:** 1 Centre for Physical Activity and Nutrition Research, Division of Sport and Recreation, Auckland University of Technology, Auckland, New Zealand

## Abstract

**Background:**

It is well established that the risk of insufficient physical activity is greater in girls than in boys, especially during the adolescent years. The promotion of active transport (AT) to and from school has been posited as a practical and convenient solution for increasing girls' total daily activity. However, there is limited information describing the associations between AT choices and girls' physical activity across a range of age, ethnic, and socioeconomic groups. The objectives of this study were to (1) investigate physical activity patterns in a large multiethnic sample of female children and adolescents, and to (2) estimate the physical activity associated with AT to and from school.

**Methods:**

A total of 1,513 girls aged 5–16 years wore sealed multiday memory (MDM) pedometers for three weekdays and two weekend days. The ethnic composition of this sample was 637 European (42.1%), 272 Pacific Island (18.0%), 207 East Asian (13.7%), 179 Maori (11.8%), 142 South Asian (9.4%), and 76 from other ethnic groups (5%). Pedometer compliance and school-related AT were assessed by questionnaire.

**Results:**

Mean weekday step counts (12,597 ± 3,630) were higher and less variable than mean weekend steps (9,528 ± 4,407). A consistent decline in daily step counts was observed with age: after adjustment for ethnicity and SES, girls in school years 9–10 achieved 2,469 (weekday) and 4,011 (weekend) fewer steps than girls in years 1–2. Daily step counts also varied by ethnicity, with Maori girls the most active and South Asian girls the least active. Overall, 44.9% of participants used AT for school-related travel. Girls who used AT to and from school averaged 1,052 more weekday steps than those who did not use AT. However, the increases in steps associated with AT were significant only in older girls (school years 5–10) and in those of Maori or European descent.

**Conclusion:**

Our data suggest that adolescent-aged girls and girls of Asian descent are priority groups for future physical activity interventions. While the apparent benefits of school-related AT vary among demographic groups, promoting AT in girls appears to be a worthwhile strategy.

## Background

Participation in regular physical activity is associated with numerous positive health outcomes in children and adolescents, including improvements to cardiovascular profiles and psychological wellbeing [1]. Conversely, insufficient physical activity is a major contributor to the burden of disease, predisposing young people to the development of obesity, diabetes, and other chronic diseases later in life. In spite of these consequences, research indicates that physical activity participation by children and adolescents continues to decline in many countries [2].

An understanding of the determinants of low physical activity in youth is essential for designing effective interventions to target those most at risk. However, obtaining objective physical activity data in a large sample can prove a challenging task. Step-counting pedometers offer a simple, cost-effective solution, providing an objective assessment of physical activity that correlates well with accelerometers [3] and observational techniques [4]. Although unable to detect the frequency or intensity of activity, the use of steps.day^-1 ^as a standard physical activity unit facilitates direct comparisons between population groups. Indeed, the practical nature of daily step count targets has increased the popularity of pedometers for quantifying physical activity in large samples and as a motivational tool for health promotion. Recent models incorporate a multiday memory (MDM) function that allows step counts on weekdays and weekend days to be analysed separately. This is an important consideration given the significant differences in physical activity behaviour between day types [5-7].

One of the most consistent observations in previous pedometry research is that girls accumulate significantly fewer steps than boys [5,8-10]. This finding suggests that girls are an at-risk group for physical inactivity, and thus, a research priority. Of particular concern are the low step counts recorded in adolescent girls, an observation preceded by a steep decline in weekday activity between childhood and adolescence [8]. During the pre-pubescent years, age-related trends are less clear. Vincent *et al.*[9] reported that weekday step counts were relatively stable with age in American, Australian, and Swedish children. While our previous observations of weekday steps in New Zealand children showed similar trends, we noted a significant decrease in weekend step counts with age [5]. Accordingly, children may experience a more pronounced reduction in steps counts with age on weekends than on weekdays. To our knowledge, no studies have assessed both weekday and weekend step counts in a large sample of children and adolescents. Participation in health behaviours such as physical activity can also vary substantially across ethnic [11] and SES groups [12]. Indeed, ethnic identity and socioeconomic status are key dimensions of health inequalities [13]. Research into the associations between pedometer steps and age also requires the potential effects of ethnicity and SES to be characterised.

Despite being largely overlooked in previous surveys of physical activity, active transport (AT) to and from school has become a popular context for increasing habitual activity in young people of all ages and physical abilities [14]. In elementary schools, for example, walking school buses have been widely adopted as a means to encourage physical activity and reduce traffic congestion [15]. Promotion of AT may be particularly beneficial for girls to accumulate steps, given that they are less likely than boys to participate in behaviours that require vigorous physical activity [7,16,17]. However, research on the impact of AT on girls daily activity levels has been equivocal. Although there is evidence supporting AT in female children [18,19] and adolescents [20], other studies have found no advantages of AT in girls [21,22]. Such discrepancies between studies may reflect differences in sample characteristics and assessment techniques. Regardless, there are currently no data quantifying the association between school-related AT and daily steps in children or adolescents.

Given recent efforts to promote activity in girls through pedometer-based interventions [23], it is essential that the correlates of daily step counts in this population group are well understood. Thus, the objectives of the present study were (1) to investigate weekday and weekend step counts in female children and adolescents from five ethnic groups, and (2) to examine the association between AT and daily steps in girls.

## Methods

### Participants

A total of 1,648 girls aged 5–16 years (mean age = 11.58 ± 2.77) were randomly selected from 39 primary (school years 1–6; ages 5–10 years), intermediate (school years 7–8; ages 11–12 years), and secondary (school years 9–10; ages 13–14 years) schools in Auckland, New Zealand. Of this initial group, 27 participants (1.6%) lost their pedometer during testing, and 108 (6.6%) provided incomplete data, resulting in a final sample size of 1,513. The ethnic composition of this sample was 637 European (42.1%), 272 Pacific Island (18.0%), 207 East Asian (13.7%), 179 Maori (11.8%), 142 South Asian (9.4%), and 76 children from other ethnic groups (5%). The East Asian ethnic group included Chinese (48.3%), Korean (29.0%), Filipino (12.1%), and other East Asian (10.6%) children; and the South Asian group included Indian (91.5%), Sri Lankan (7.0%), Bangladeshi (0.7%), and Nepalese (0.7%) children. Socioeconomic status (SES) was estimated using the Ministry of Education decile classification system for New Zealand schools. Participants from schools with a decile rating of 1–3 were categorised into the 'Low' SES group, while those from schools rated 4–7 and 8–10 were considered 'Middle' and 'High', respectively. Ethical approval for this study was obtained from the Auckland University of Technology Ethics Committee. Written informed consent was provided by each participant and her legal guardian.

### Physical activity

Daily physical activity was assessed using the New Lifestyles NL-2000 (Lee's Summit, MO) MDM pedometer. The NL-2000 provides similar accuracy and better precision [24] than the widely used Yamax Digiwalker series while offering the additional benefits of a MDM function, whereby step counts are automatically recorded each day for seven days. Each NL-2000 pedometer was checked for defects prior to use in the study by observing the recorded step count after walking 100 paces [9]. Instrumental error did not exceed 3% in any of the pedometers. Approximately 40 children per week were tested between the months of August, 2004 and August, 2005 (excluding school holidays). Participants were given a short explanation about the study and a demonstration on how to attach a pre-sealed pedometer to the waistline, and were instructed to wear their pedometer all day for seven consecutive days (except when sleeping or swimming). On the seventh day of testing, researchers visited participants at their school to collect the devices and record the number of steps taken on each day. This resulted in a maximum of five full days of data (three weekdays and two weekend days).

Intermediate and Secondary school participants were given a compliance questionnaire at the time of pedometer collection to record any times the device was not worn during the monitoring period. The low reliability of self-report techniques in children [25] necessitated the use of a proxy compliance questionnaire in elementary school participants. Parents/caregivers of participants less than ten years of age completed a questionnaire the night before the pedometer was due for collection to assess compliance outside the school environment. Non-compliance of elementary children during school hours was considered negligible due to active teacher assistance. Participants who removed their pedometer for more than one hour on any day had the steps accumulated on that day removed from analysis. Additionally, participants with more than one weekday and one weekend lost due to incomplete data were excluded from the final data set. Finally, daily step counts below 1,000 or above 30,000 were regarded as outliers to allow for the possibility that non-compliant individuals were not identified [26]. Information on AT patterns was collected by questionnaire. Intermediate and secondary school participants were asked which travel modes they usually used to travel to and from school (both routes assessed separately), and the parents/caregivers of participants at elementary school completed an equivalent proxy questionnaire.

### Statistical analyses

Data were analysed using SPSS version 12.0.1 for Windows (SPSS Inc., Chicago, IL). Differences in participant characteristics (age, SES) among ethnic groups were assessed by two-way ANOVA and significant associations were examined by pairwise comparisons using *t*-tests. One-way ANOVA and Bonferroni *post hoc *tests were used to determine where significant differences in step counts existed among ethnic, age, socioeconomic, and AT groups. Associations among weekday and weekend step counts, ethnicity, SES, and school year group were assessed using factorial repeated measures ANOVA. Chi squared analysis was used to examine significant differences in the frequency of using active transport modes to travel to/from school across ethnic, SES, and school year groups. A *P *value less than 0.05 was used to indicate statistical significance.

## Results

Age differences were present among the ethnic groups (*P *< 0.001), with East Asian girls significantly older (12.33 ± 2.43) than European (11.31 ± 2.92) and Maori (11.32 ± 2.60) girls. The average decile of Pacific Island girls was lower than that all other ethnic groups, followed by Maori, South Asian, Other, East Asian, and European girls. In addition, the mean decile varied by school year group (*P *< 0.001), with year 9–10 girls averaging the highest decile (6.87 ± 3.51) and year 7–8 girls averaging the lowest decile (4.48 ± 2.4). The frequencies of the different ethnic groups also varied significantly across school year levels (*P *< 0.001). However, no significant differences in pedometer compliance were detected across ethnic (*P *= 0.228), age (*P *= 0.394), or SES (*P *= 0.105) groups.

Table 1 gives the mean weekday and weekend step counts for the present sample grouped according to ethnicity, school year group, and SES. Mean weekday steps were consistently higher and tended to have smaller standard deviations than mean weekend steps across all subgroups. However, the magnitude of the difference between weekday and weekend steps varied significantly by school year group and SES (*P *< 0.001). In particular, the mean difference in step counts between day types tended to be greater for older girls and for those from lower SES groups. There were significant differences in weekday steps among the ethnic groups: Maori girls were the most active, followed by Pacific Islanders, and then Europeans. For weekend activity, Maori and European girls averaged the highest step counts, followed by Pacific Islanders. South and East Asian girls were the least active ethnic groups during weekdays and the weekend. Both weekday and weekend step counts decreased with school year group. Average weekday steps were highest for girls from the middle SES group, followed by the low, and the high groups. However, girls from the middle and high SES groups were more active than girls from the low SES group during weekends.

**Table 1:** Pedometer-determined physical activity (steps.day^-1^)

	Weekday Steps		Weekend Steps	
	*N*	Mean ± SD	*N*	Mean ± SD
Total	1447	12597 ± 3630	1355	9528 ± 4407
Ethnicity* **				
European	617	12830 ± 3486	580	10433 ± 4175
East Asian	196	11121 ± 3203	185	7599 ± 3730
South Asian	136	11314 ± 3089	136	7673 ± 3224
Maori	172	14096 ± 3764	150	10868 ± 5410
Pacific Island	254	12992 ± 3973	235	9189 ± 4749
Other	72	12071 ± 3203	69	8988 ± 3146
School Yr * **				
1–2	161	14572 ± 2714	150	12512 ± 4109
3–4	196	14394 ± 3377	187	11309 ± 4384
5–6	200	13566 ± 3529	194	10027 ± 4079
7–8	231	11882 ± 3288	222	7514 ± 3050
9–10	659	11537 ± 3567	602	8812 ± 4423
Socioeconomic status* **				
Low	565	12711 ± 3738	524	8724 ± 4406
Middle	259	13405 ± 3581	253	10410 ± 4422
High	623	12157 ± 3487	578	9871 ± 4289

* Significantly different for weekday steps (*P *< 0.005).

** Significantly different for weekend steps (*P *< 0.005).

To investigate the interactions among the key demographic factors associated with activity in this sample, ethnicity (European, Maori, Pacific Island, East Asian, and South Asian), school year group (1–2, 3–4, 5–6, 7–8, and 9–10), SES (high, medium, and low) and day (weekday and weekend) were entered into a 5 × 5 × 3 × 2 factorial repeated measures ANOVA (Ethnicity by School Year by SES by Day; Table 2). Mean step counts differed significantly between weekdays and weekends, with significant interactions between day and school year group, and day and SES, but not between day and ethnicity. This indicates that the significant decrease in activity observed on weekend days varies by school year group and SES, but not by ethnicity. Analysis of the between subject variance revealed a significant association between overall mean step count and both school year and ethnicity, with a significant interaction between school year and SES. Inspection of the estimated marginal means for the latter interaction revealed that the distribution of mean steps across SES groups was noticeably different between years 1–2 (Low SES: 13,508, Middle SES: 12,434, High SES: 14,597) and years 9–10 (9,474, 11,563, 9,758), but was similar for the other school year groups.

**Table 2:** Results of a factorial repeated measures ANOVA (Ethnicity by School Year Group by SES)

Source	*F*	*P*
Within Subjects		
Day	176.123	0.000**
Day × Ethnicity	0.546	0.742
Day × School Year Group	2.960	0.019*
Day × SES	6.414	0.002*
Day × Ethnicity × School Year Group	1.231	0.219
Day × Ethnicity × SES	1.830	0.051
Day × School Year Group × SES	0.997	0.432
Day × Ethnicity × School Year Group × SES	0.981	0.501
Between Subjects		
Ethnicity	8.425	0.000**
School Year Group	17.416	0.000**
SES	1.046	0.352
Ethnicity × School Year Group	1.015	0.440
Ethnicity × SES	0.878	0.553
School Year Group × SES	2.290	0.026*
Ethnicity × School Year Group × SES	1.097	0.323

*Significant (*P *< 0.05) level

**Significant (*P *< 0.005) level

**Figure 1:**
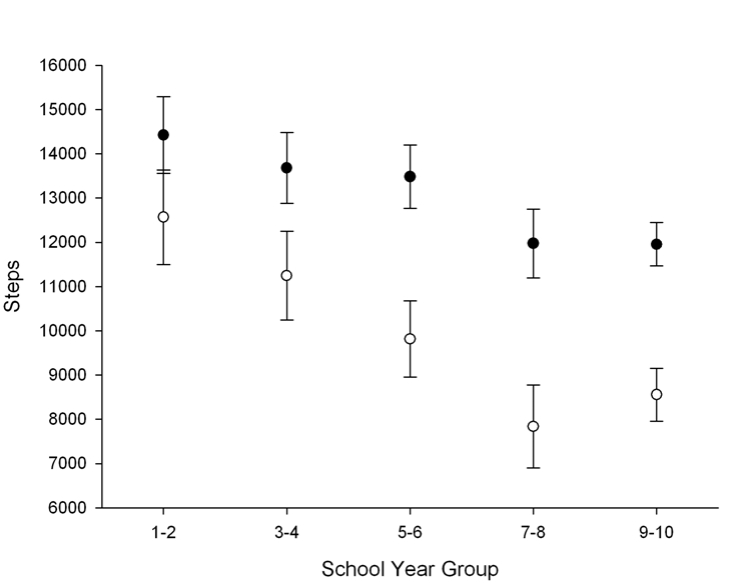
**Physical activity during weekdays (●) and weekends (○) vs. school year group (adjusted for ethnicity and SES)**. *Significantly lower than the previous school year group for weekend steps (P < 0.05).

Figure 1 shows the pedometer-determined physical activity of girls during weekdays and weekends according to school year group when adjusted for differences in ethnic and SES group. Weekend steps were consistently lower than weekday steps across all the school year groups. With increasing school year group, there was a tendency for both weekday and weekend steps to decrease. On average, girls in years 9–10 accumulated 2,469 less steps on weekdays (95% CI: 1,112, 3,826; *P *< 0.001), and 4,011 less steps on weekends (95% CI: 2,345, 5,677; *P *< 0.001) than girls in years 1–2. The steepest decline in activity for adjacent groups occurred between school years 5–6 and 7–8, with the latter group averaging 1,549 fewer steps on weekdays (95% CI: 16, 3,002; *P *= 0.052) and 1,983 fewer steps on weekend days (95% CI: 179, 3,780; *P *= 0.024) than their younger counterparts. The increase in weekday and weekend steps between years 7–8 and years 9–10 was not significant.

**Figure 2:**
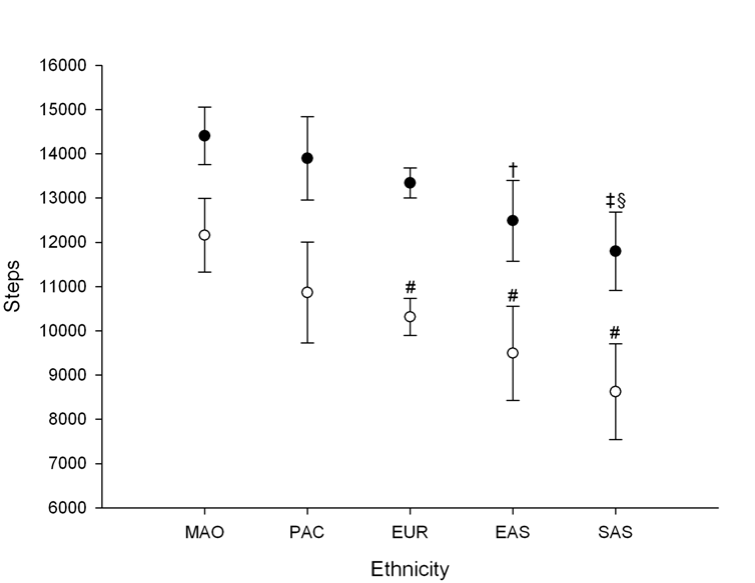
**Physical activity during weekdays (●) and weekends (○) vs. ethnic group (adjusted for school year group and SES)**. †Significantly lower than MAO for weekday steps (P < 0.05). ‡Significantly lower than MAO for weekday steps (P < 0.005). §Significantly lower than PAC and EUR for weekday steps (P < 0.05). #Significantly lower than MAO for weekend steps (P < 0.005).

Figure 2 shows the pedometer-determined physical activity of girls from five ethnic groups on weekday and weekend days after adjustment for differences in school year group and SES. Girls from all ethnic groups achieved significantly lower step counts on weekend days than on weekdays. South Asian girls accumulated the least activity on weekdays and the weekend, averaging 11,797 (95% CI: 10,882, 12,685) steps and 8,623 (95% CI: 7,541, 9,706) steps, respectively. In comparison, Maori girls had the highest step counts, accumulating an average of 14,407 (95% CI: 13,758, 15,056) steps on weekdays, and 12,159 (95% CI: 11,329, 12,989) on weekends.

Overall, 44.9% of the girls in this study used AT modes to travel at least one way when commuting to and from school. Of this group, 69.4% actively transported both to and from school, and 30.6% actively transported one way only. Walking was the most common mode of AT (97.3%), followed by cycling (2.7%). The frequency of AT groups varied significantly across ethnicities (*P *< 0.001), school year groups (*P *= 0.001), and SES groups (*P *< 0.001). Of all the ethnic groups, Maori were the most likely to use AT to travel either one or both ways to and from school (55.0%). Frequencies of AT for other ethnic groups were relatively similar, ranging from 41.5% (Pacific Island) to 47.4% (South Asian). Across school year groups, the frequency of AT steadily increased from years 1–2 (39.8%) until years 7–8 (52.9%), before decreasing at years 9–10 (42.9%). Girls belonging to the low or medium SES groups were also significantly more likely to use AT (47.7% and 46.2%, respectively) than those belonging to the high SES group (41.8%).

Multifactor ANOVA revealed significant differences in daily weekday steps among AT groups after adjustment for SES, school year, and ethnicity (*P *< 0.001). Girls that used AT both to and from school averaged 1,052 more weekday steps (95% CI: 284, 1,821; *P *= 0.001) than those who did not use AT. While the difference in steps between girls who used AT one way only and those who did not use AT was similar in magnitude (834), the relatively low number of girls that use AT one way reduced the statistical power of the comparison (95% CI: -177, 1,845; *P *= 0.092). Significant interactions were observed between AT and ethnicity (*P *= 0.038), and between AT and school year group (*P *= 0.003), but not between AT and SES (*P *= 0.472). This indicates that the difference in weekday steps between AT groups varies with ethnicity and school year group, but not SES group. Identical analysis for daily weekend steps showed no significant association with AT (*P *= 0.550): girls that used AT both to and from school and those who used AT one way only averaged 280 (95% CI: -572, 1,132; *P *= 1.000) more and 261 (95% CI: -1,458, 937; *P *= 1.000) fewer weekend steps (respectively) than girls that did not use AT.

**Figure 3:**
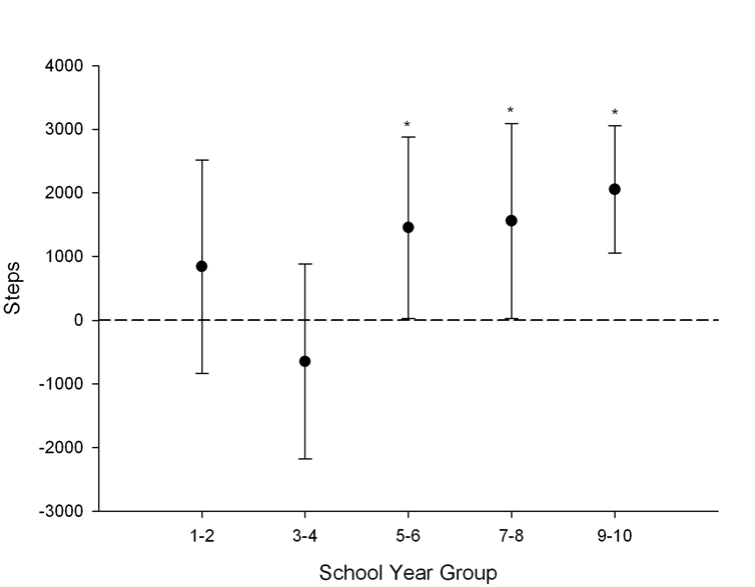
**Differences in weekday activity between AT and non-AT girls grouped by school year (adjusted for ethnicity and SES)**. *Significantly higher mean step counts in AT girls than in non-AT girls.

**Figure 4:**
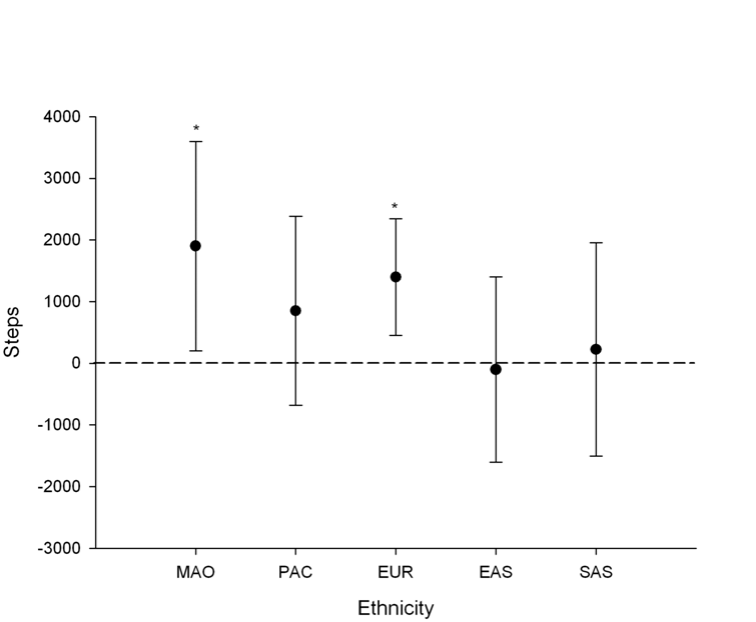
**Differences in weekday activity between AT and non-AT girls grouped by ethnicity (adjusted for school year group and SES)**. *Significantly higher mean step counts in AT girls than in non-AT girls.

Figures 3 and 4 show the mean difference in daily weekday steps between AT groups (AT to and from school – no AT) across school year groups (after adjustment for ethnicity and SES), and ethnic groups (after adjustment for school year and SES). For girls in years 1–4 there were no significant differences in weekday step counts between AT groups. However, older girls that used AT to travel to and from school achieved significantly more steps on weekdays than those that did not use AT. The largest difference in weekday steps between these AT groups was observed for girls in years 9–10, who averaged 2,053 more steps (95% CI: 1,049, 3,057). This was followed by girls in years 7–8 who averaged 1,559 more steps (95% CI: 27, 3,091), and girls in years 5–6, who averaged 1,455 more steps (95% CI: 33, 2,877). There were no significant step count differences for girls that used AT for one trip when compared to those that did not use AT. When analysed according to ethnic group, only Maori and European girls had significant differences in weekday steps counts between AT groups. Maori girls that used AT to travel to and from school averaged 1,900 (13.9%) more steps (95% CI: 202, 3,598), and European girls averaged 1,398 (11.0%) more steps (95% CI: 453, 2,344) than those from the same ethnicity that did not use AT to travel either to or from school.

## Discussion

The first objective of this study was to examine weekday and weekend step counts in a multiethnic sample of female children and adolescents. Several key patterns were identified that characterise the physical activity behaviours of girls and thereby provide a basis for developing strategies to promote activity in this population. The most consistent finding was that girls accumulated considerably fewer steps on weekends than on weekdays. This pattern was evident for all demographic groups, with the greatest step count difference between day types observed for older girls and those from lower SES groups. While differences in activity between day types have been observed using both pedometers [5] and other objective measurement techniques [6,7], comparisons of weekday and weekend activity in diverse samples are limited. The present study provides evidence that the effect of day type on girls' activity is significant across a range of ethnic, school year, and SES groups, and thus, provides support for widespread initiatives to encourage activity during the weekend.

With increasing school year, an inverse relationship with mean steps was observed. Interestingly, the age-related decline in steps was more pronounced for weekends than weekdays. Between years 1–2 and 9–10, our results suggest that population daily step counts for New Zealand girls would decrease by 8.2–25.0% on weekdays and by 20.4–41.8% on weekend days (95% CI). Within this age range, the steepest decline in activity occurred between primary and intermediate schools (years 5–6 and years 7–8), corresponding to a likely population decrease of 0.1–21.1% on weekdays and 2.0–35.4% on weekend days. Similarly, in a sample of 1,046 American girls aged 6–18 years, Le Masurier et al [8] reported that weekday pedometer steps were relatively constant during the elementary years before falling off in school years 7–12. Taken together, these findings suggest that the transition from primary to intermediate school may be an important intervention period for arresting the decline in school-centred activity. It is also possible that the onset of puberty triggers a physiological mechanism for conserving energy that contributes to a reduction in physical activity. In any case, the steady decrease in weekend step counts observed between school years 1–8 highlights the need for physical activity in the home environment to be promoted from an early age.

Given New Zealand's ethnic diversity and the substantial health inequalities across different ethnic groups [13], it is imperative to determine ethnic variation when assessing health risk factors like physical activity. A major strength of this study was the ability to compare activity behaviours across five diverse ethnic groups. Girls from Asian ethnic groups accumulated fewer steps than all other ethnic groups, with South Asian girls of particular concern. The development of tailored physical activity interventions for motivating Asian girls in a culturally appropriate manner may help to redress these imbalances. The findings from the present study in children and adolescents extend results from our previous research in children, where we noted that Asian boys and girls aged 5–12 years accumulated fewer steps than their European, Pacific, or Maori counterparts [5]. Earlier studies investigating the influence of ethnic background on physical activity suggest that white adolescents are generally more active than ethnic minorities, with less consistent results for children [27]. Vincent *et al.*[9] also reported significant differences in weekday step counts between children from America, Australia, and Sweden. Clearly, representation of all major ethnic groups within a chosen population is necessary when characterising pedometer determined physical activity. Furthermore, there is a need for more in depth research into underlying cultural and social determinants of physical activity behaviours.

The second objective of this study was to evaluate AT practices in New Zealand girls. Although active commuting to and from school provides an opportunity for children and adolescents of all physical abilities to engage in physical activity on a regular basis [14], less than half of the present sample regularly used AT modes for school-related travel. Particularly low utilisation rates were reported for school years 1–2, possibly due to the perceived risks of allowing young girls to walk unsupervised, and the logistics of accompanied walking given the high prevalence of working parents [28]. Between years 7–8 and years 9–10, a marked decline in AT rates was observed. This decease may reflect the transition from intermediate to secondary schools, the latter having larger catchment areas which may preclude some girls from walking the distance to/from school. In addition, girls from high socioeconomic backgrounds were less likely to utilise AT than those from middle or low SES groups. These results are in accordance with an earlier study in Auckland school children that reported comparatively lower rates of walking home from school among children from the higher socioeconomic stratums [29]. Interestingly, the latter authors found no differences in parental perceptions of risk among the various sociodemographic groups. While there is evidence that underprivileged families have less access to motorised transport [29], socioeconomic differences in parental employment obligations and social norms may also be contributing factors. Reasons for the relatively high AT participation among Maori girls are also unclear, but may be related to the overrepresentation of Maori in low SES regions [28], and/or the importance placed on physical activity participation in this ethnic group.

Recent efforts to promote activity among young people by way of school-related AT are based on the assumption that walking to and from school results in meaningful increases in daily physical activity. Pedometers are the ideal instrument to assess such changes given the ambulatory nature of this transport mode. Our data indicate that for a given SES, school year, and ethnicity, using AT modes to commute both to and from school would be associated with a population increase of 2.2–14.4% steps for New Zealand girls. Estimates of the change in weekday steps between girls who do not use AT and those who use it either to or from school are similar but less precise (-1.4–14.6%). Overall, walking to and from school appears to be an effective means for girls to accumulate steps on weekdays. These findings are in agreement with most [18,20,19] but not all [21,22] previous investigations of AT in young people. In any case, it is likely that the availability of AT for all school children (regardless of their physical fitness or demographic group), coupled with the ensuing environmental benefits of reduced traffic congestion and lower carbon dioxide emissions, will continue to drive the popularity of initiatives encouraging active commuting to and from school.

While AT was associated with higher weekday step counts overall, the strength of the association varied according to school year group and ethnicity. For example, while we would expect AT to and from school to increase population steps anywhere from 0.3% to 28.7% in girls above year 4 (allowing for differences in SES and ethnicity), for younger girls the true benefit of AT is less clear, ranging from -15.0% to 18.0%. The trend towards a stronger relationship between step counts and AT with increasing school year may reflect older girls walking further to/from school than younger girls, either because of greater distances to commute or less parental restrictions on distances travelled by AT. Similarly, our results suggest that AT would be associated with positive changes to weekday steps for Maori and European girls, with more variable outcomes for Pacific Island, East Asian, and South Asian girls. Such findings highlight the importance of assessing activity behaviour within demographic subgroups, and suggest that tailoring AT initiatives to the groups likely to experience the greatest benefits may be worthwhile. However, it should be noted that the 95% confidence intervals for the change in steps with AT were relatively wide in most instances. Thus, AT may be advantageous even in groups that showed no significant associations with weekday steps.

It has previously been hypothesised that AT in children may potentiate physical activity participation. Cooper *et al.*[21] reported that AT was associated with elevated physical activity throughout the school day in boys. Conversely, it is possible that active children are more likely to walk to/from school than inactive children, and that AT is an indicator rather than a cause of higher activity levels. The cross-sectional design of our study precludes establishment of the causative effects of behavioural variables such as AT. However, the lack of significant step count differences between AT groups on weekends (non-AT days) would suggest that overall activity levels have little or no influence on AT participation, and that any extra weekday physical activity as a result of AT does not persist into the weekend. Clearly, more study is needed to realise the potential downstream benefits of school-related AT.

It should be noted that potential predictors of AT participation (such as distance from school, seasonal variation, availability of motorised transport, and parental employment obligations etc) were not examined. Adjustment for differences in the distance from school, in particular, may offset the observed variation in the association between AT and physical activity across ethnic and school year groups (or reveal significant variation across SES groups). In addition, the applicability of the present results to children in other countries is uncertain. Identifying the potential differences in cultural or societal influences on physical activity behaviour between populations requires further study.

## Conclusion

In summary, this study provides the first investigation of weekday and weekend step counts in female children and adolescents. The low number of steps accumulated on weekends suggests that physical activity promotion in the home environment is a priority. Furthermore, the low physical activity levels of older school girls warrant attention, as do those of South Asian girls. AT to and from school appears to be an effective approach for increasing weekday step counts in girls, although the activity benefits for young girls and Asian ethnic groups are unclear. Together, these findings highlight the importance of assessing activity behaviour across a range of demographic groups, and provide impetus for tailoring initiatives to the groups most likely to benefit.

## Abbreviations

ANOVA: Analysis of variance;

AT: Active transport;

SES: Socioeconomic status;

MDM: Multiday memory.

## Competing interests

The author(s) declare that they have no competing interests.

## Authors' contributions

All authors were involved in the conception and design of the study. EKD and JSD were responsible for data collection and statistical analyses. All authors contributed to writing and approved the final manuscript.

## References

[B1] US Department of Health and Human Services (1996). Physical Activity and Health: A Report of the Surgeon General.

[B2] Dollman J, Norton K, Norton L (2005). Evidence for secular trends in children's physical activity behaviour. Br J Sports Med.

[B3] Kilanowski CK, Consalvi AR, Epstein LH (1999). Validation of an electronic pedometer for measurement of physical activity in children. Pediatr Exerc Sci.

[B4] Scruggs PW, Beveridge SK, Eisenman PA, Watson DL, Shultz BB, Ransdell LB (2003). Quantifying physical activity via pedometry in elementary physical education. Med Sci Sports Exerc.

[B5] Duncan JS, Schofield G, Duncan EK (2006). Pedometer-determined physical activity and body composition in New Zealand children. Med Sci Sports Exerc.

[B6] Gavarry O, Giacomoni M, Bernard T, Seymat M, Falgairette G (2003). Habitual physical activity in children and adolescents during school and free days. Med Sci Sports Exerc.

[B7] Jago R, Anderson CB, Baranowski T, Watson K (2005). Adolescent patterns of physical activity differences by gender, day, and time of day. Am J Prev Med.

[B8] Le Masurier GC, Beighle A, Corbin CB, Darst PW, Morgan C, Pangrazi RP, Wilde B, Vincent SD (2005). Pedometer-determined physical activity levels of youth. J Phys Act Health.

[B9] Vincent SD, Pangrazi RP, Raustorp A, Michaud Tomson L, Cuddihy TF (2003). Activity Levels and Body Mass Index of Children in the United States, Sweden, and Australia. Med Sci Sports Exerc.

[B10] Wilde BE, Corbin CB, Le Masurier GC (2004). Free-living pedometer step counts of high school students. Pediatr Exerc Sci.

[B11] Gordon-Larsen P, McMurray RG, Popkin BM (1999). Adolescent physical activity and inactivity vary by ethnicity: The National Longitudinal Study of Adolescent Health. J Pediatr.

[B12] Sallis JF, Zakarian JM, Hovell MF, Hofstetter CR (1996). Ethnic, socioeconomic, and sex differences in physical activity among adolescents. J Clin Epidemiol.

[B13] Ministry of Health (2002). Reducing Inequalities in Health.

[B14] Tudor-Locke C, Ainsworth BE, Popkin BM (2001). Active commuting to school: an overlooked source of childrens' physical activity?. Sports Med.

[B15] Kingham S, Ussher S (2005). Ticket to a sustainable future: An evaluation of the long-term durability of the Walking School Bus programme in Christchurch, New Zealand. Transp Pol.

[B16] Trost SG, Pate RR, Sallis JF, Freedson PS, Taylor WC, Dowda M, Sirard J (2002). Age and gender differences in objectively measured physical activity in youth. Med Sci Sports Exerc.

[B17] Sleap M, Warburton P (1996). Physical activity levels of 5-11-year-old children in England: cumulative evidence from three direct observation studies. Int J Sports Med.

[B18] Cooper AR, Andersen LB, Wedderkopp N, Page AS, Froberg K (2005). Physical activity levels of children who walk, cycle, or are driven to school. Am J Prev Med.

[B19] Page A, Cooper AR, Stamatakis E, Foster LJ, Crowne EC, Sabin M, Shield JP (2005). Physical activity patterns in nonobese and obese children assessed using minute-by-minute accelerometry. Int J Obes.

[B20] Tudor-Locke C, Ainsworth BE, Adair LS, Popkin BM (2003). Objective physical activity of filipino youth stratified for commuting mode to school. Med Sci Sports Exerc.

[B21] Cooper AR, Page AS, Foster LJ, Qahwaji D (2003). Commuting to school: are children who walk more physically active?. Am J Prev Med.

[B22] Metcalf B, Voss L, Jeffery A, Perkins J, Wilkin T (2004). Physical activity cost of the school run: impact on schoolchildren of being driven to school (EarlyBird 22). BMJ.

[B23] Schofield L, Mummery WK, Schofield G (2005). Effects of a controlled pedometer-intervention trial for low-active adolescent girls. Med Sci Sports Exerc.

[B24] Duncan JS, Schofield G, Duncan EK, Hinckson EA (2007). Effects of age, walking speed, and body composition on pedometer accuracy in children. Res Q Exerc Sport.

[B25] Sallis JF, Buono MJ, Roby JJ, Micale FG, Nelson JA (1993). Seven-day recall and other physical activity self-reports in children and adolescents. Med Sci Sports Exerc.

[B26] Rowe DA, Mahar MT, Raedeke TD, Lore J (2004). Measuring physical activity in children with pedometers: Reliability, reactivity, and replacement of missing data. Pediatr Exerc Sci.

[B27] Sallis JF, Prochaska JJ, Taylor WC (2000). A review of correlates of physical activity of children and adolescents. Med Sci Sports Exerc.

[B28] Statistics New Zealand (2002). 2001 Census Data.

[B29] Roberts I, Norton R (1994). Auckland children's exposure to risk as pedestrians. N Z Med J.

